# Durable superhydrophobic surfaces made by intensely connecting a bipolar top layer to the substrate with a middle connecting layer

**DOI:** 10.1038/s41598-017-10030-9

**Published:** 2017-08-30

**Authors:** Jinghui Zhi, Li-Zhi Zhang

**Affiliations:** 1 Key Laboratory of Enhanced Heat Transfer and Energy Conservation of Education Ministry, School of Chemistry and Chemical Engineering, South China University of Technology, Guangzhou, 510640 China; 2 State Key Laboratory of Subtropical Building Science, South China University of Technology, Guangzhou, 510640 China

## Abstract

This study reported a simple fabrication method for a durable superhydrophobic surface. The superhydrophobic top layer of the durable superhydrophobic surface was connected intensely to the substrate through a middle connecting layer. Glycidoxypropyltrimethoxysilane (KH-560) after hydrolysis was used to obtain a hydrophilic middle connecting layer. It could be adhered to the hydrophilic substrate by covalent bonds. Ring-open reaction with octadecylamine let the KH-560 middle layer form a net-like structure. The net-like sturcture would then encompass and station the silica particles that were used to form the coarse micro structures, intensely to increase the durability. The top hydrophobic layer with nano-structures was formed on the KH-560 middle layer. It was obtained by a bipolar nano-silica solution modified by hexamethyldisilazane (HMDS). This layer was connected to the middle layer intensely by the polar Si hydroxy groups, while the non-polar methyl groups on the surface, accompanied by the micro and nano structures, made the surface rather hydrophobic. The covalently interfacial interactions between the substrate and the middle layer, and between the middle layer and the top layer, strengthened the durability of the superhydrophobic surface. The abrasion test results showed that the superhydrophobic surface could bear 180 abrasion cycles on 1200 CW sandpaper under 2 kPa applied pressure.

## Introduction

In recent decades, the non-wettability phenomenon of surfaces, such as the Lotus plant leaves and water strider legs, were categorized as one of the hot topics for many researchers and tremendous works^1–4^. Superhydrophobic surfaces are defined as non-wettability surfaces whose water contact angles are larger than 150° and their rolling angles are less than 10°. They can be used in a wide range of technological applications, such as, self-cleaning^5, 6^, anti-corrosion^2, 7^, oil-water separation^8, 9^, anti-fouling^10, 11^ and so on. Up to now, it has been well recognized that the ways to fabricate superhydrophobic surfaces rely on two crucial aspects: micro/nano-scaled hierarchical structures and low surface energy materials^12, 13^. So there are two kinds of methods to fabricate superhydrophobic surfaces. The one is to make micro-nano hierarchical surfaces modified subsequently with low surface energy materials, and the other one is to build a micro-nano roughness with low surface energy materials directly^14^. Considerable efforts have been devoted to preparing superhydrophobic materials for various purposes. However, regardless of the fabrication processes, the durability of superhydrophobic surfaces is still a severe obstacle to their practical uses in industries and everyday life^15, 16^.

Due to the abrasion from external forces, the superhydrophobicity of fabricated surfaces is easy to lose. This is the main reason for durability problems. So more and more researchers begin to focus on how to increase the mechanical durability and wear resistance. Many different strategies have been proposed to enhance the durability of superhydrophobic surfaces. In order to avoid the micro/nanostructures being destroyed by contact pressure upon load and to prevent the superhydrophobic materials from losing the superhydrophobicity, Men *et al*. reported a smooth liquid-repellent surface^17^. It could display non-wetting behavior towards water and many organic liquids. Meanwhile it could bear the abrasion of filter paper under 500 g weight for a while. However, the contact angles of the liquid on the non-wetting surface were just about 60 degree. The superiority of Cassie-Baster state was not fully realized. Wong *et al*. proposed a method to fabricate ultradurable superhydrophobic surface. It provided a rapid and substrate-independent approach for ultradurable surface with abrasion, chemical and UV-resistance^18^. Xue *et al*. investigated a robust, self-healing superhydrophobic surface by coating polydimethylsiloxane and octadecylamine (ODA) on poly(ethylene terephthalate) (PET) fibers^19^. When the superhydrophobic surface suffered abrasion by external forces, the self-roughening property of ODA could be easily restored. So the rough structures could be healed automatically and the superhydrophobicity could be recovered to some extent. Qu *et al*. employed organosilane surface-functionalized quarts sand particles to fabricate durable superhydrophobic materials^14^. Among the quarts sand particles, covalent bonds were formed with the help of condensation by hydroxyl groups. The mechanical durability property of the superhydrophobic materials was enhanced. However, when the materials were coated on substrates, covalent bonds were not formed between the substrate and the material, which makes the coating easy to lose from the substrate when abraded. Santhosh Kumar *et al*. reported a robust superhydrophobic material by grafting long alkyl chains on silica nanoparticle surface^20^. However, as bulk superhydrophobic material, the hardness was not noticed. Cui *et al*. prepared highly durable superhydrophobic surfaces through painting epoxy resin on substrates followed by a three-step method subsequently^21^. In the work of Figueira reported, in order to delay the corrosion of materials and metallic structures, they employed the hybrid sol-gel coatings based on glycidoxypropyl-trimethoxysilane (KH-560) to protect substrates against corrosion. The coating could form covalent bonds with the substrates to enhance the mechanical abrasion resistance^22^.

Even though the aforementioned progress, the durability is still a problem. As seen, to increase the mechanical durability, the connecting between the coating materials and the substrates, as well as the connecting between the micro/nano structures and the coating materials, should be strengthened simultaneously. However previous researches did not solve this problem thoroughly. In order to make the superhydrophobic surfaces more durable, this paper proposes a new approach: (1) a transitional middle connecting layer which is hydrophilic is used to build covalent bonds with the substrate. (2) A layer of bi-polar nano-silica particles which have both Si hydroxy groups and Si methyl groups, is made and then coated on the middle layer. This top layer is connected to the middle layer intensely by the covalent bonds built by the polar Si hydroxy groups. The non-polar Si methyl groups of the bi-polar nano-silica particles, accompanied by the micro and nano structures, make the surface superhydrophobic. In this way, the different parts of the coating are connected intensely. The coating is also connected to the substrate intensely. The prepared superhydrophobic surfaces in this method possess high static contact angles of about 165° and small rolled off angles of about 1°. Through abrasion test, the results show that the superhydrophobic surfaces can bear 180 abrasion cycles under 2 kPa pressure on 1200 CW sandpaper. When the superhydrophobic surfaces are soaked in 3.5 wt% NaCl solution, the Cassie-Baxter state can be kept for more than 20 days.

## Experimental

### Materials

Glass sheets (75 × 25 × 1 mm) were bought from Guangzhou Qianhui Co., China. Tetraethoxysilica (TEOS) and acetone were obtained from Guangzhou Jinke Co., China. Hydrogen peroxide (H_2_O_2_, 30%), sulfuric acid (H_2_SO_4_, 95.0%~98.0%) and NaCl solid particles were purchased from Guangzhou Qianhui Co., China. Glycidoxypropyltrimethoxysilane (KH-560, C_9_H_20_O_5_Si) was purchased from Dow Corning. Hexamethyldisilazane (HMDS), octadecylamine (ODA), hydrophobic silica (50~100 nm), hydrophilic silica (7~ 40 nm) were all supplied by Aladdin, Shanghai, China. All the reagents used in this paper were not purified further. High purity water (HPW) was prepared by a Purescience water purification system.

### Fabrication of precursor solution

The solution to make the middle connecting layer was prepared as follows. 40 ml ethanol was added into a beaker. 2.5 ml KH-560 was dripped into the ethanol solution and mixed by vigorously stirring for 1 h. Then 1 ml high purity water and 0.8 g ODA were introduced into the solution, respectively. After 30 min mechanical stirring, 0.5 g hydrophobic silica were dropped into the mixture. With at least 2 h constant stirring, the mixture formed the middle connecting layer solution. And in the whole process, the temperature should be kept at 30 °C.

The method to make the bi-polar nano-silica solution was as follows. 4.7 ml hexamethyldisilazane (HMDS) and 0.15 g hydrophilic silica (7~40 nm) were added into 10 ml ethanol with mechanical stirring for 30 min at room temperature. Methyl groups of HMDS were grafted on the silica particles to replace some of the hydroxyl groups on the hydrophilic silica.

### Fabrication of superhydrophobic surface with middle connecting layer

Before the fabrication of the superhydrophobic surface on the glass sheet, the glass substrate was modified by hydroxyl with piranha solution (3:1mixture of 98% H_2_SO_4_ and 30% H_2_O_2_)^23^. The glass substrate was put in the 70 °C piranha solution for 100 min, which could make the substrate hydroxylate fully. Then the glass was washed by high purity water for several minutes and dried in oven subsequently. After that, the glass was coated by the precursor solution made previously. Firstly, the middle connecting layer precursor solution was coated onto the treated glass slide with drop-coating method. Then, the coated surface was put in a stove at 80 °C for 40 min. Secondly, the coated surface with the middle connecting layer was dropped with the bi-polar nano-silica solution. Then, the sample was kept at 140 °C for 90 min. After that, the prepared superhydrophobic surface was taken out of the oven. Different investigations proceeded.

### Characterization

The wettability of the prepared superhydrophobic surfaces was measured by Dataphysics OCA 2.0 contact angle system at ambient temperature with a 9 μl water droplet. The rolled off angle were measured by a drop of water released onto the inclined substrate from a defined height. The minimum angle of the inclined surface at which the drop completely rolling off the surface was recorded and that was the rolled off angle^24^. The rolling-off processes of the water droplet on the superhydrophobic surfaces were recorded by a high-speed camera (pco. Dimax HD, CooKe) with a frame rate of 6000/s. Each kind of sample was measured 5 times on different positions and the average value was used. The morphological structures of the samples were observed using scanning electron microscope (SEM, Merlin, LEO1530VP, Germany). Platinum was sprayed onto the samples before observing the morphology in order to enhance the conductivity. Energy dispersion spectroscopy (EDS, Inca400, Oxford, England) techniques were used to obtain the chemical compositions of the modified samples. The samples were magnified 1000 times in EDS measurements. Attenuated total internal reflectance infrared (ATR-IR) spectrometry (BRUKER550, Germany) was performed at a resolution of 4 cm^−1^ with 64 scans over the range of 4000–400 cm^−1^ to measure the possible groups on the films. The durability of the Cassie-Baxter state was tested through soaking the film in mimetic sea water (3.5 wt% NaCl solution). Additionally, the mechanical durability of the sample was evaluated by adhesive tape peeling test and a sandpaper-abrasion method.

## Results and Discussion

### Super-hydrophobicity

**Figure 1:**
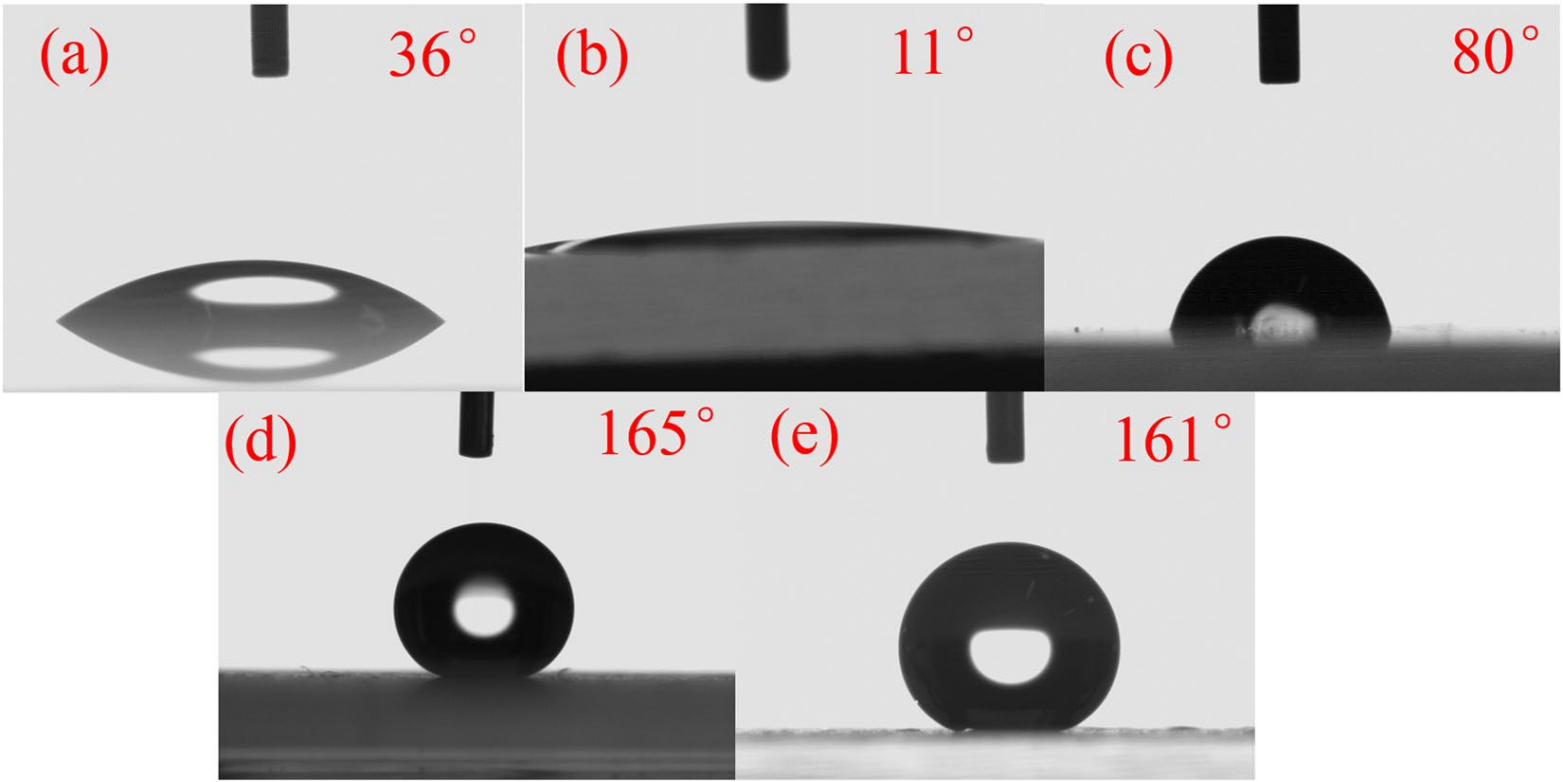
Contact angle of a water droplet on the surface of (**a**): a base glass; (**b**): hydroxylated glass; (**c**): the middle connecting layer coated on the substrate; (**d**): obtained superhydrophobic surface; (**e**): the superhydrophobic surface after soaked in mimetic sea water for half a month.

**Figure 2:**
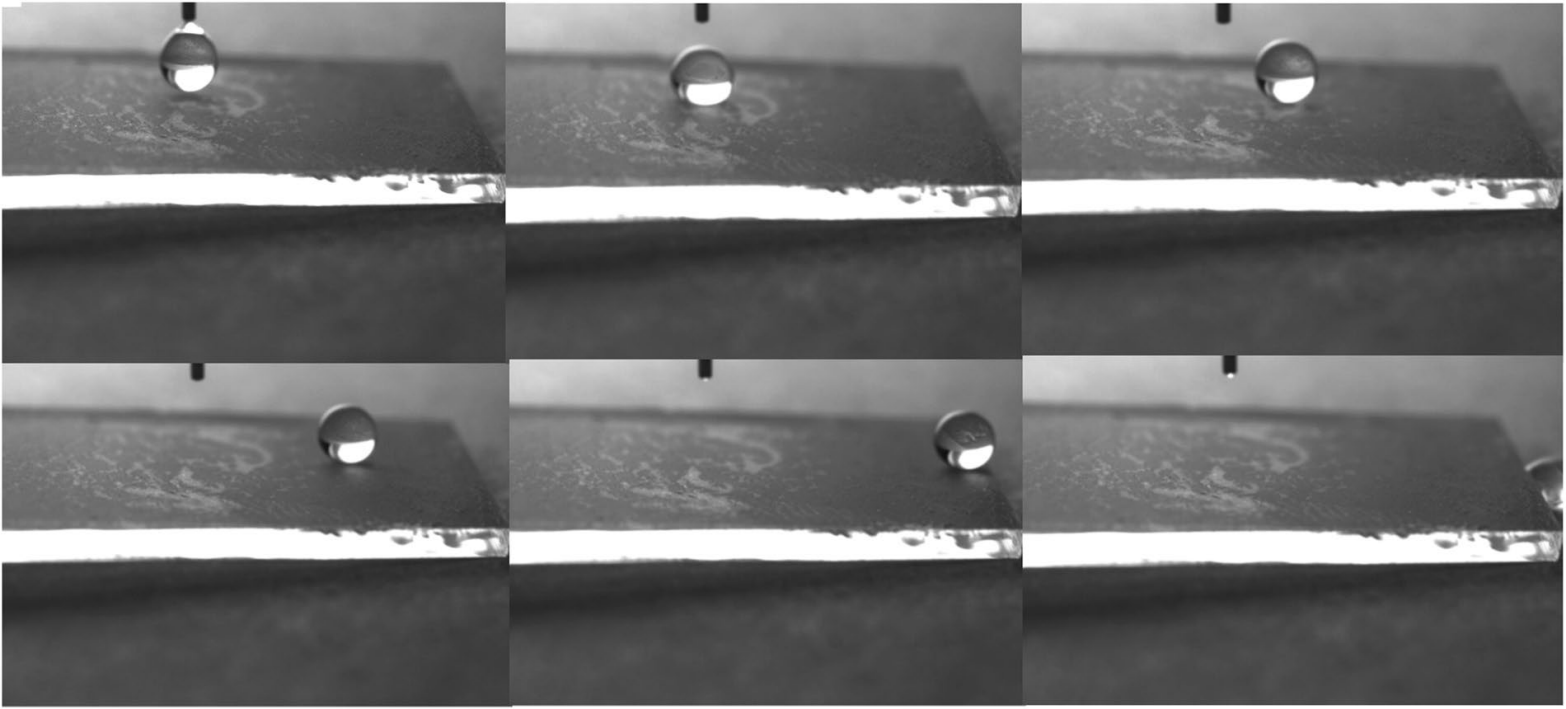
The rolling-off processes of a 6 µL water droplet on the prepared superhydrophobic surfaces.

The wettability of the obtained superhydrophobic surface was tested by Dataphysics OCA 2.0 contact angle system at ambient temperature with a 9 μl droplet. When the water droplet was dropped on the surface, the contact angle could be up to 165° which was showed in Fig. 1(d). In Fig. 1(b), the contact angle of the hydroxylated glass substrate was showed. The contact angle was just 11°, which was lower than the contact angle (39°, showed in Fig. 1(a)) of the base glass. It indicated that the hydroxyl groups were successfully grafted on the base glass. This was important for the covalent bonds formed between the glass substrate and the middle connecting layer. The contact angle of the hydrophilic middle connecting layer was also displayed in Fig. 1(c). The angle was 80°. A superhydrophobic surface required not only a high static contact angle, but also a low adhesion and a low friction to water droplet^25^. So a high-speed camera (pco. Dimax HD, CooKe) with a frame rate of 6000/s was used to capture the rolling-off processes of the 6 μl droplet on the superhydrophobic surfaces. The minimum inclined angle at which the water rolling off was the rolled off angle of the superhydrophobic surface. Figure 2 displayed the rolling-off processes. As could be seen from Fig. 2, the water droplet was rolling off from the obtained superhydrophobic surface easily when the surface was inclined just with 1°. So the rolled off angle of the superhydrophobic surface was just about 1°. This was attributed to the low energy of the modified surface^26^. Meanwhile, it also indicated that the bipolar silica was successful made. Figure 1(e) showed the contact angle of the superhydrophobic surface soaked in mimetic sea water for half a month, which will be discussed later.

### Durability

**Figure 3:**
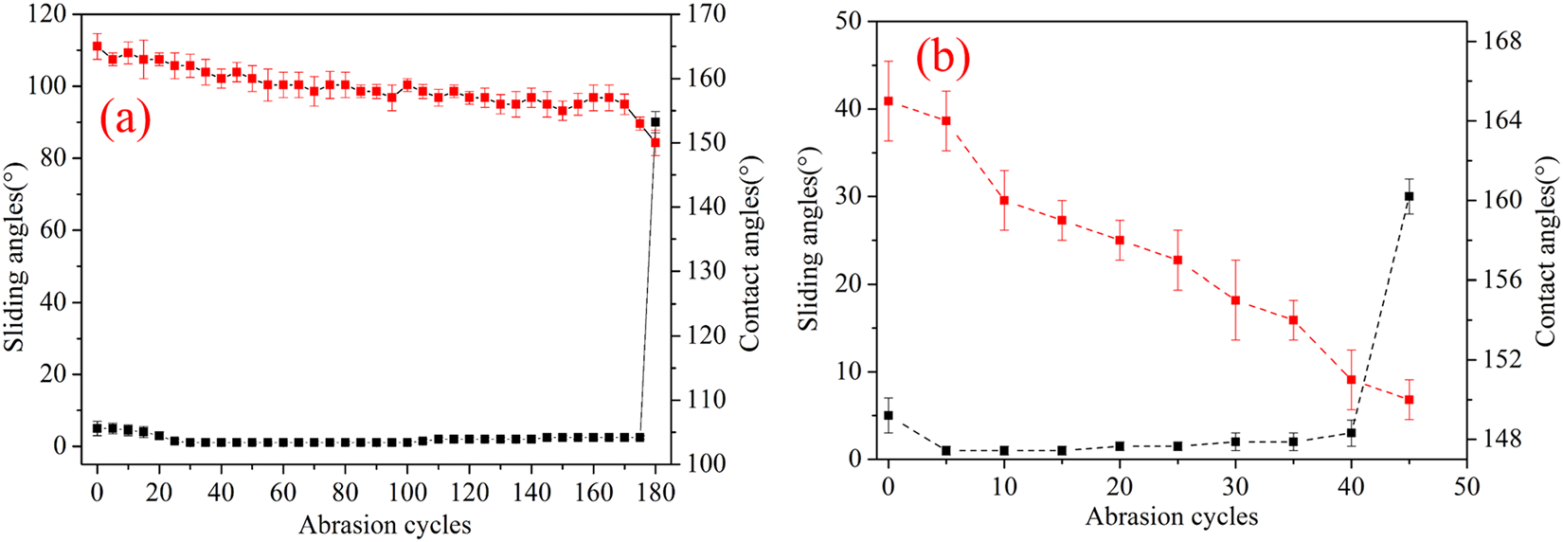
Wettability of the superhydrophobic surfaces versus abrasion cycles (**a**): 2 kPa pressure on the superhydrophobic surface (the red dotted line was the contact angle, and the black one was the rolled off angle); (**b**): 3 kPa pressure on the superhydrophobic surface.

The mechanical durability of the obtained superhydrophobic surface was measured by a sandpaper-abrasion experiment^27–29^. Figure 3 showed the change in wettability of the superhydrophobic surfaces versus the abrasion cycles. In Fig. 3(a), the abrasion test was carried out on a 1200 CW sandpaper under 2 kPa applied pressure. The coated surface of glass substrate was faced to the sandpaper. The abrasion distance of one cycle was 17 cm. Under the impetus of force, substrates moved slowly along with the ruler. The contact angles and the rolled off angles were tested after every five abrasion cycles. Each test was measured 5 times on different positions of a surface. The average values were used. After 180 cycles of abrasion test, the static contact angle of the superhydrophobic surface changed from 165° to 150° gradually. However, the rolled off angles was kept at < 5°during the abrasion tests. It then suddenly exceeded 90° when the abrasion cycles was greater than 180. This indicated that the non-polar methyl groups were successfully grafted on the top layer to lower its surface energy. The top bipolar layer was connected to the middle layer tightly. Possible explanation for the tight connecting is that covalent bonds linking the substrate and the coating were formed. Figure 3(b) displayed another abrasion test which was carried out on a 1200 CW sandpaper under 3 kPa applied pressures over the coated glass substrate. The abrasion distance was still 17 cm. The trend of change in rolled off angles was similar to that in Fig. 3(a). In contrast, the durability of the superhydrophobic surface which was only coated with a bipolar nano-silica layer was tested under 2 kPa pressure on 1200 CW sandpaper. However, it could only bear 1 cycle abrasion, whose durability was much less than the superhydrophobic surface with the middle connecting layer. This result indicated that the middle connecting layer played an important role in connecting the glass substrate and the bipolar nano-silica layer.

**Figure 4:**
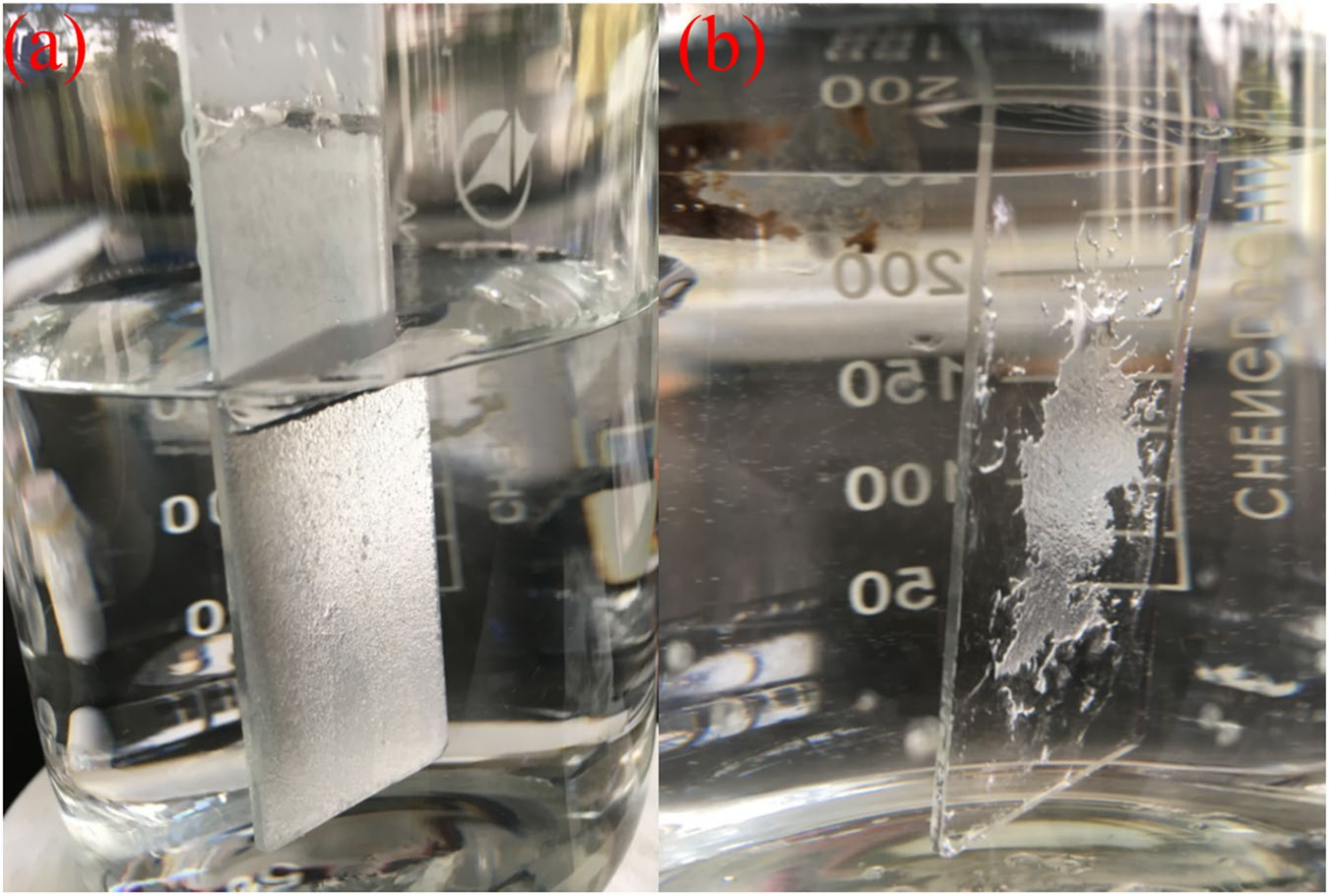
Immersion of the prepared superhydrophobic surface into mimetic sea water (**a**): the superhydrophobic surface with the middle connecting layer; (**b**): the superhydrophobic surface just coated with a bipolar nano-silica layer without the middle connecting layer.

As known, when the superhydrophobic surface was in Cassie-Baxter state, the surface could possess special properties, such as self-cleaning, anti-smudge, corrosion resistance and so on^30–32^. The Cassie-Baxter state was more durable, the superhydrophobicity property of the surface could kept longer. In order to investigate the durability of the Cassie-Baxter state of the superhydrophobic surface, the coated glass substrates were immersed into mimetic sea water (3.5 wt% NaCl solution), as seen from Fig. 4. From Fig. 4(a), it could be seen that when the superhydrophobic surface with the middle connecting layer was soaked into the solution, it showed a mirror-like phenomenon. This could be attributed to the existence of trapped air between water and the superhydrophobic surface at Cassie-Baxter state^33, 34^. The superhydrophobicity of the surface in the mimetic sea water could be kept for more than 20 days. The contact angle of the superhydrophobic surface was still kept at 161° (the picture of the contact angle was showed in Fig. 1 (d)). However, the surface only coated with the bipolar nano-silica layer without the middle connecting layer could not bear the mimetic sea water immersing. After five minutes, the superhydrophobic film fell off from the glass substrate, as showed in Fig. 4(b). The superhydrophobicity was lost. This indicated that the middle connecting layer has led to the intense connecting between the glass substrate and the bipolar nano-silica layer. So the durable superhydrophobic surface was formed. The superhydrophobicity of the superhydrophobic surface could keep for a long time.

**Figure 5:**
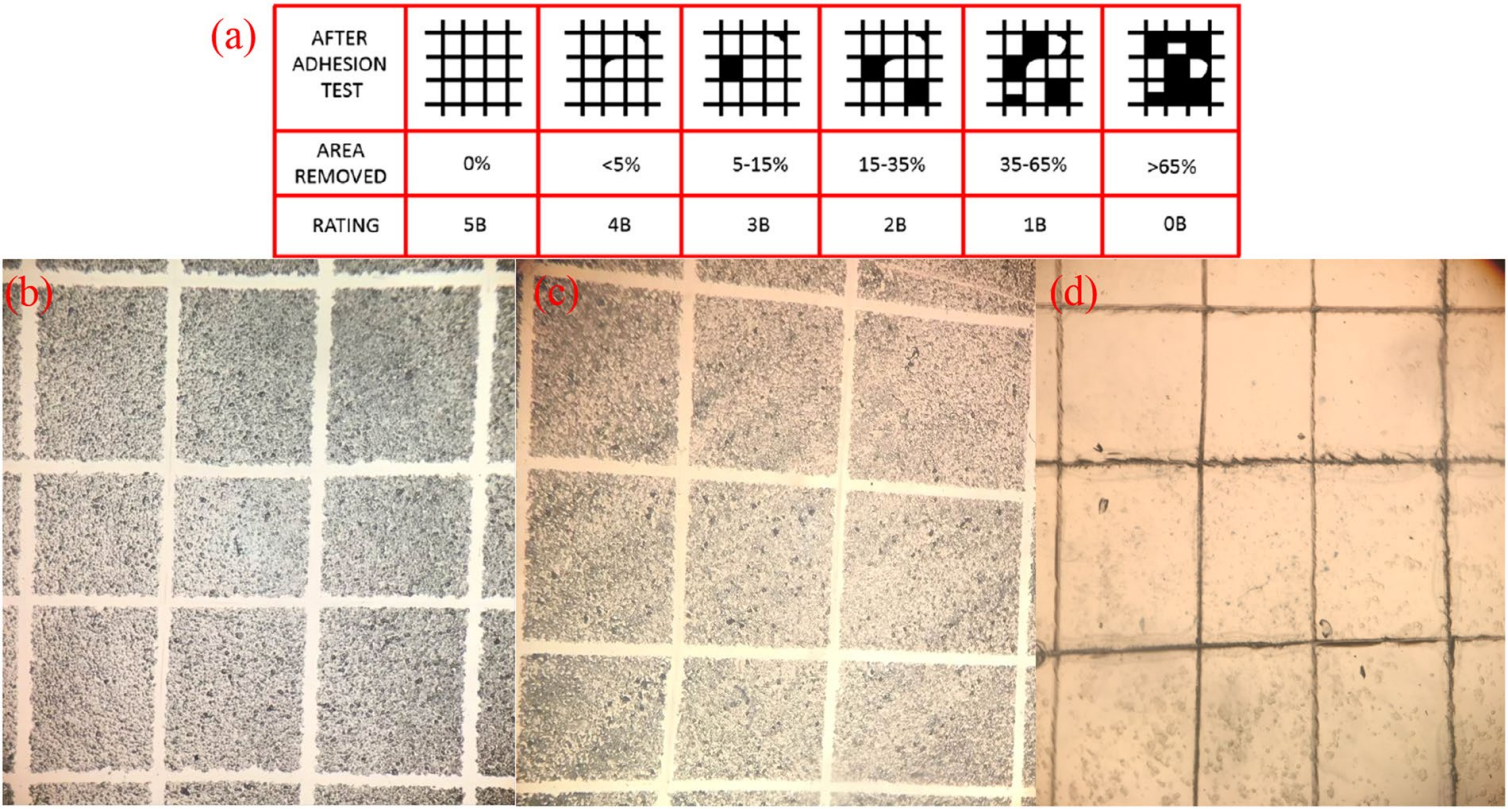
Cross-hatch tape adhesion test for prepared coatings (**a**) Indicative chart of adhesion ranking, according to the ASTM D3359 (reproduced from 34); the black part was the area removed. (**b**): coated only by the middle hydrophilic film and tested; (**c**) coated by the hydrophilic-hydrophobic bipolar composite film and tested; (**d**): coated only by the top bipolar layer, without the middle layer.

Subsequently, the durability of the fabricated superhydrophobic surface was also tested by cross-hatch tape adhesion, as showed in Fig. 5. Tape peeling was a material removal test that had been used to test the adhesion strength of non-wettable coatings to substrates^35^. A tape was applied on the tested surface and pressed in order to ensure that there was no air entrapment and the entire adhesive surface was in contact with the non-wettable surface. Then the tape was peeled off from one end. Figure 5(a) is an indicative chart of adhesion rankings according to the ASTM D3359 (reproduced from ref. 34). 5B, 4B, 3B, 2B, 1B and 0B were the grades used to quantify the extent of the adhesion of the coated film on the substrate. When the coated film was rated as 5B, it meant that the film exhibited the highest adhesion to a substrate. When it was rated 0B, it meant the poorest adhesion. Figure 5(b) displayed the adhesion test of the middle connecting layer. It was seen that the transitional middle layer was rated as 4B. Figure 5(c) showed the adhesion test of the top bipolar layer coated on the middle layer. It was also rated as 4B. These indicated that the top bipolar layer exhibited a strong adhesion to the middle connecting layer. Meanwhile the middle layer also had a strong adhesion to the substrate. Figure 5(d) showed the adhesion test of the top bipolar layer coated on the glass substrate without the middle layer. It could be seen that the coating materials were easily removed from the substrate after the adhesion test. The grade was rated as 0B. This testified the use of the transitional middle layer in connecting both the top bipolar layer and the substrate. During adhesive tape test, the applied pressure was 1 kPa.

**Figure 6:**
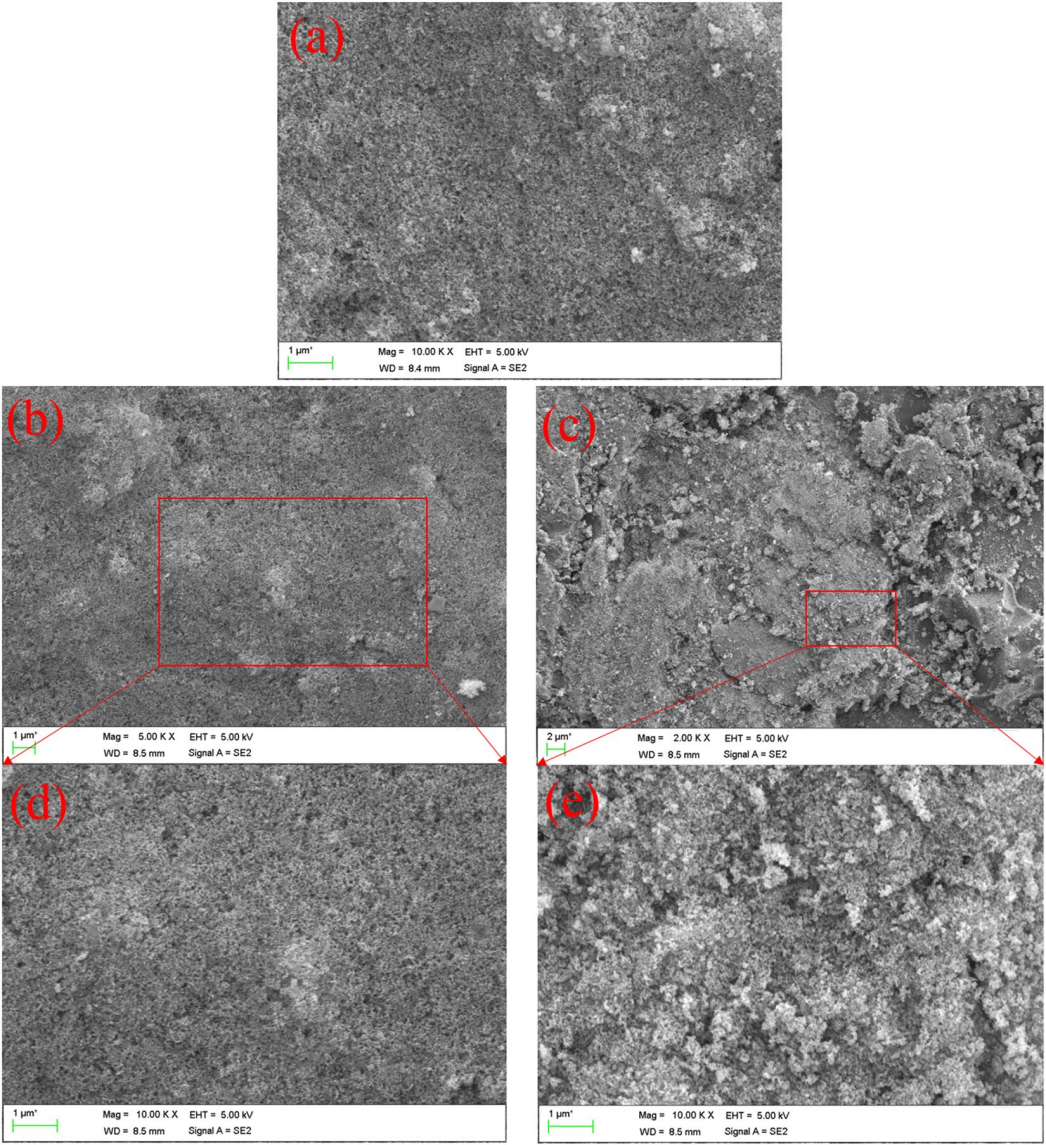
The SEM images of the superhydrophobic surface before and after the durability tests (**a**): the superhydrophobic surface without test; (**b**): The superhydrophobic surface after 16 days soaked in NaCl (3.5 wt%) solution; (**c**): The superhydrophobic surface after 70 cycles abrasion tests; (**d**) the magnification image of (**b**); (**e**) the magnification image of (**c**)

In order to verify the changes of the chemical composition of the superhydrophobic surface after the various durability tests, EDS detection was performed. The results were listed in Table 1. From Table 1, it could be seen that the percentages of O and Si atoms were reduced after 120 cycles abrasion tests, comparing to the superhydrophobic surfaces without any tests. This indicated that after the continuous abrasion the covalent bonds were broken and the bipolar nanosilica layer was peeled off from the middle connecting layer. The SEM test in Fig. 6(c) also verified this. From Fig. 6(c), it showed that some nanosilicas were disappeared. After the superhydrophobic surface was soaked in NaCl (3.5 wt %) solution for 16 days, the percentages of the atoms on the surface changed slightly (the added atoms of Na and Cl were from NaCl solution). It manifested that the durability of bipolar nanosilica layer was not affected by the NaCl (3.5 wt %) solution. The unchanged morphology in Fig. 6(b) also proved the strong durability of the obtained superhydrophobic surface.

**Table 1:** Changes of the chemical composition of the superhydrophobic surface before and after different durable test.

	without any test^1^		70 cycles abrasion^2^		16 days soaked^3^	
	wt%	atom%^4^	wt%	atom%^4^	wt%	atom%^4^
C	21.08	30.95	46.44	58.72	24.05	34.42
O	41.08	45.29	30.18	28.64	41.45	44.53
Si	37.85	23.77	23.38	12.64	33.10	20.25
Na					0.46	0.34
Cl					0.94	0.46

^1^The superhydrophobic surface without any test.

^2^The superhydrophobic surface after 70 cycles abrasion tests.

^3^The superhydrophobic surface after 16 days soaked in NaCl (3.5wt%) solution.

^4^The percentage of atom.

### The connecting between the middle layer and the substrate

**Figure 7:**
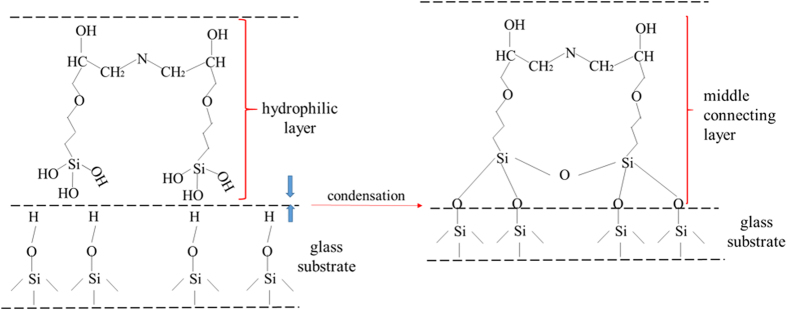
Schematic illustration of the process of covalent bond formed between the substrate and middle transitional layer.

In order to achieve high mechanical durability, the rough structures should be robust and the low surface energy material should be durable^16, 36^. For this objective, in this work, a new approach was proposed: (1) a transitional middle connecting layer was used to build covalent bonds with the hydroxylated substrate. The 3D net-like structure of the transition layer encompassed and stationed the hydrophobic silica particles to form coarse micro structures. The durability was increased. (2) A layer of bi-polar silica nano particles which had both Si hydroxy groups and Si methyl groups, was made and coated on the middle layer. This top layer was connected to the middle connecting layer intensely by the covalent bonds built by the polar Si hydroxy groups. While the non-polar Si methyl groups on the surface, accompanied by the micro and nano structures, made the surface rather hydrophobic. Figure 7 schematically showed the process of covalent bonds formed between the substrate and the transitional middle layer. As the KH-560 possessed strong mechanical property and excellent adhesion to substrate^37, 38^, it was chosen as the transitional middle layer. During the hydrolysis of KH-560 in ethanol, the three –SiOCH_3_ groups transformed into three Si-OH groups. When the solution was coated on the cleaned glass substrate, the Si-OH group would react with the –OH group on the glass surface at heat treatment condition, as Fig. 7 showed. The Si-O-Si covalent bonds were formed. The connection was strengthened between the glass substrate and the middle connecting layer. Meanwhile, the opening-ring reaction of epoxy rings in KH-560 with the amine groups in ODA could result in a 3D crosslink network structure^39^. The primary amine contained in the ODA was active. When the primary amine met epoxy groups, ring-open reaction would happen and a net-like structure would be formed. KH-560 contained epoxy groups. So the ODA and KH-560 would react.

**Figure 8:**
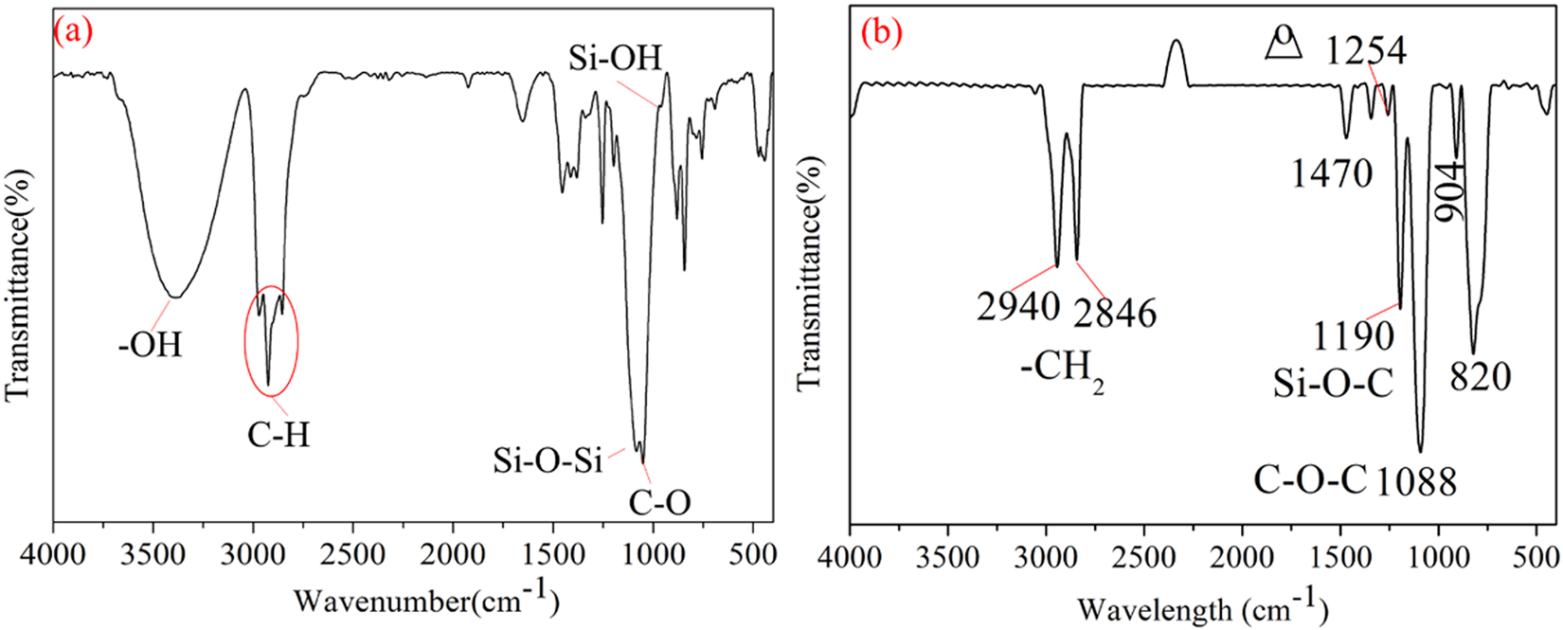
ATR-IR spectra analysis of (**a**): the middle connecting film without hydrophobic silica; (**b**): the KH-560 film.

For the sake of verifying the covalent bonds formed in the preparing process, the ATR-IR spectras of the middle connecting film was showed in Fig. 8(a). In order to accurately confirm whether or not the covalent bonds of Si-O-Si were formed between the substrate and the middle layer, the hydrophobic silica particles were not added in the middle layer solution during the ATR-IR spectras test process. The surface coated by the middle layer solution was cured at 80 °C for 40 min and then 140 °C for 90 min. After the curing process, the formed middle layer on the glass substrate was tested by ATR-IR spectra. The base line of the ATR-IR spectra was tested by using cleared glass substrate. From Fig. 8(a), the Si-OH at 970 cm^−1^ indicated that the hydrolyzing reaction happened. The Si-OH groups formed during the hydrolyzing reactions would get covalently bonded with the glass substrate by the formation of Si-O-Si bonds^17^. The Si-O-Si peak at 1150 cm^−1^ was observed in Fig. 8(a), which indicated that the covalent bonds were formed between the glass substrate and the middle connecting layer. It also could be seen that the peaks of N-H at 3300 cm^−1^ and 1555 cm^−1^ disappeared^40^ and the characteristic peak of the epoxy groups around 912 cm^−1^ was also vanished. However, the peak of 3400 cm^−1^ signified the presence of –OH groups. These changes of peaks indicated that the opening-ring reaction had been occurred between the KH-560 and ODA. Figure 8(b) showed the ATR-IR spectra of the KH-560 film. From the ATR-IR spectra, it could be seen that there were no –OH peaks at 3400 cm^−1^ and Si-O-Si peaks at 1150 cm^−1^. The peaks of epoxy group at 1254 cm^−1^ were detected. These further testified the reaction of KH-560 and ODA, and the covalent bonds formed between the middle connecting layer with the glass substrate.

### The connecting between the middle layer and the top bipolar layer

**Figure 9:**
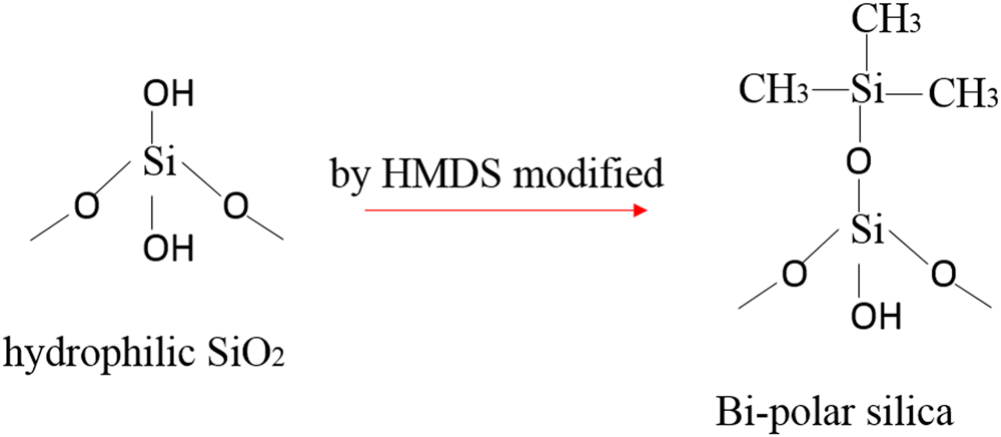
Schematic illustration of the process of the bipolar silica formed in the top bipolar layer solution.

**Figure 10:**
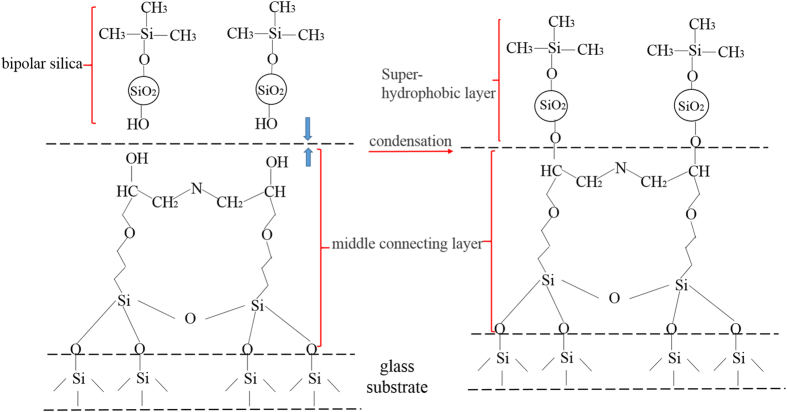
Schematic illustration of the process of covalent bonds formed between middle layer and the top bipolar layer.

In order to enhance the strong connection between the middle layer and the hydrophobic film to resistant external wearing, bipolar materials was needed. In this research, bipolar silica which possessed both Si hydroxy groups and Si methyl groups was prepared, as showed in Fig. 9. Hydrophilic nano-silica particles were modified by HMDS to acquire –Si-(CH_3_)_3_ groups on the silica surface^41^. The –Si-(CH_3_)_3_ groups replaced part of –OH groups and the bipolar silica was formed. When the top bipolar layer solution was coated on the middle layer, the residual –OH groups on the bipolar silica surface occurred dehydrating reaction with the –OH groups that were formed in the opening-ring reaction of epoxy rings in KH-560 with the amine groups in ODA during the previous heat treatment. The covalent bonds were formed, as displayed in Fig. 10. It illustrated the presence of covalent bonds formed between the middle layer and the top bipolar layer. So the ODA and KH-560 would react. As Fig. 10 showed, the 3D cross-link network structure was formed. This 3D crosslink network structure could encompass and station the silica solid particles to obtain robust rough structures of high mechanical durability. In consideration of the strong non-polarity of the hydrophobic silica particles, it was chosen in this work in order to avoid the reaction with the hydroxyl formed during the reaction of KH-560 and the ODA. So the possibility to form covalent bonds between the middle connecting layer and the bi-polar nanoparticle layer could be enhanced further.

**Figure 11:**
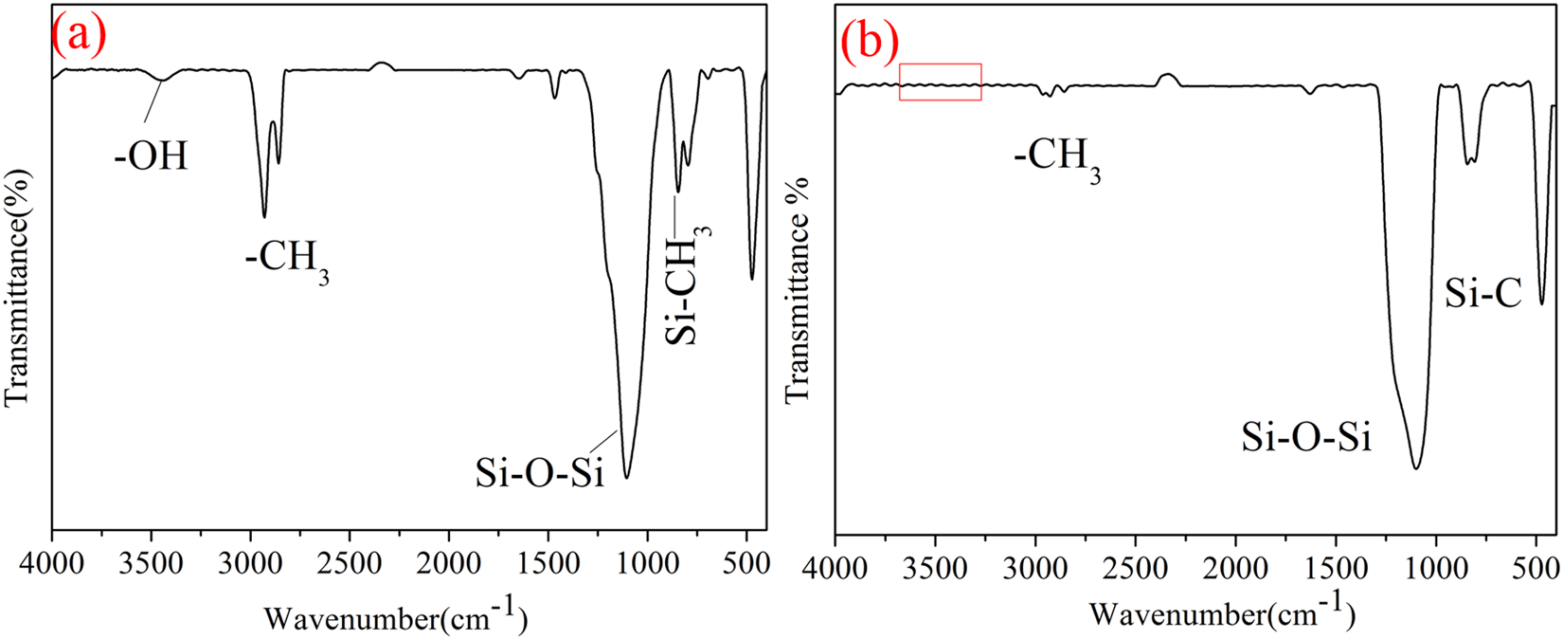
ATR-IR spectra analysis of (**a**): top bipolar layer solution; (**b**): the top bipolar layer coated on the middle layer.

In order to verify the covalent bonds formed between the middle layer and the top bipolar layer in the preparing process, the ATR-IR spectra of the top bipolar layer solution and the coating of top bipolar layer coated on the middle layer were displayed in Fig. 11. Before the ATR-IR spectra test of the top bipolar layer solution, the solution was dropped on a sheet of KBr. The sheet was dried under ultraviolet light. The base line was used pure KBr sheet. The top bipolar layer solution was coated on the middle layer and dried at 140 °C for 90 min. Then the formed coating was also tested by ATR-IR spectra. The base line of the coating of ATR-IR spectra was tested by using cleared glass substrate. In Fig. 11 (a), the characteristic peaks of -CH_3_ were appeared around 2957 cm^−1^ and the Si-CH_3_ peaks were found around 840 cm^−1^ ^42^. The feature peaks of –OH were also found around 3400 cm^−1^. These occurred peaks manifested that the hydrophilic silica was successfully modified by HMDS and the bipolar silica was formed. However, in Fig. 11(b), the feature peaks of –OH around 3400 cm^−1^ were disappeared, which might be attributed to the formation of covalent bonds between the top bipolar layer and the middle layer.

### The morphological study of the superhydrophobic surface

**Figure 12:**
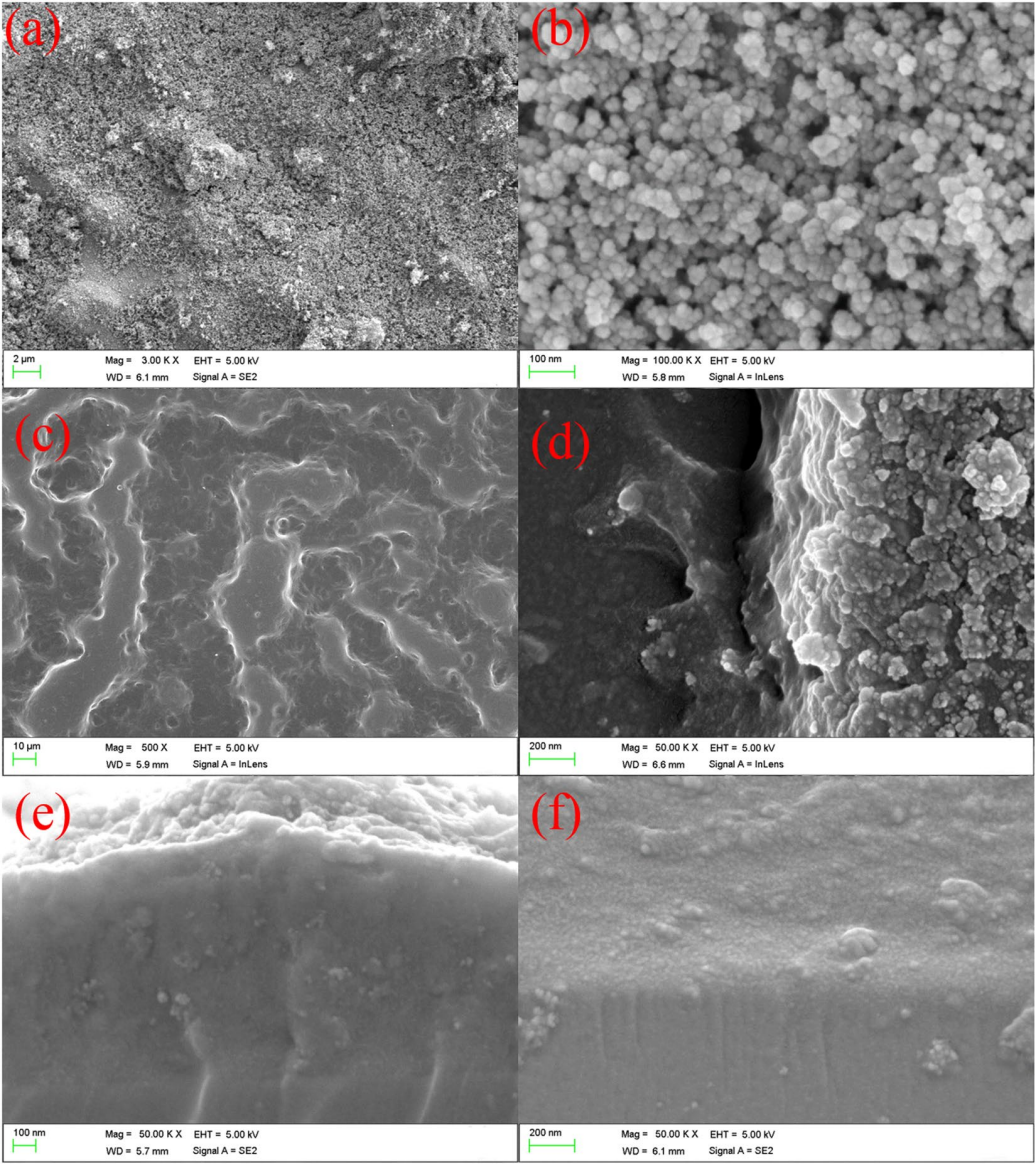
SEM images of (**a**): the top surface of the whole superhydrophobic film; (**b**): enlarged picture of (**a**); (**c**): top surface, after only the hydrophilic middle layer was coated; (**d**): surface photo inside the middle layer showing the silica solid particles were wrapped by the 3D network structure formed by KH-560 and ODA; (**e**): the cross section of the whole superhydrophobic film; (**f**) the cross section of the middle connecting layer on the substrate.

In order to observe the morphology of the obtained superhydrophobic surface, high magnified SEM images were taken. Figure 12 showed the SEM images of the top bipolar layer, the middle connecting layer and the cross section of the superhydrophobic surface. From Fig. 12(a) and (b), it clearly displayed that the top bipolar layer of the whole superhydrophobic film contained silica nanoparticles. The silica particles were bounded tightly together because of the Si-O-Si covalent bonds. The covalent bonds could increase the durability of the low surface energy material. In Fig. 12(c) and (d) were shown the morphologies of the middle connecting layer. Figure 12(c) was the top surface of the hydrophilic middle layer without the coating by the top layer. Microstructure was formed to enhance the mechanical durability^43^. Figure 12(d) was the inside structure of the middle connecting layer, which was taken when some thickness of the middle layer was abraded off. It showed that the silica solid particles were wrapped by the 3D-network structure formed by KH-560 and ODA. This could intensify the robustness of the rough structures. For the sake of investigating the interfacial connections between the substrate and the middle connecting layer, and the middle connecting layer and the top bipolar layer, the cross-sectional morphologies were also examined by SEM, as listed in Fig. 12(e) and (f). As could be seen from Fig. 12(e) and (f), the middle connecting layer was tightly bonded to the top of the glass substrate. No split at the interface was observed. Such integrated structure should be attributed to the formed covalent bonds between the glass substrate and the middle connecting layer during the curing process at high temperature. Similarly, the transitional middle layer and the top bipolar layer also formed integrated structure by covalent bonds. This integrated structure has greatly strengthened the durability of the prepared superhydrophobic surfaces.

The thickness was observed. The thickness of the middle connecting layer was about 800~1000 nm. The thickness of the bi-polar nano-silica layer was about 200 nm. The thickness of the middle connecting layer and the bi-polar nano-silica layer was observed from the cross sections of the whole superhydrophobic layer displayed in Fig. 12(e). After continuous abrasion, the covalent bonds were broken and the connecting force was weakened. As a result, the coating was lost from the surface. When all the bi-polar nano-silica layer was lost from the middle connecting layer, the non-wettability was disappeared and the superhydrophobicity was lost. After that, the thickness of the coating was about 700 nm, which was the residual hydrophilic middle connecting layer.

## Conclusions

In summary, we demonstrated a mechanically durable superhydrophobic surface which was prepared through intensely connecting a bipolar top layer to the substrate with a middle connecting layer. The middle connecting layer was obtained with the hydrolysis reaction of glycidoxypropyltrimethoxysilane (KH-560) and the opening ring reaction of KH-560 with octadecylamine (ODA). The bipolar top layer was achieved through the partial modification of hydrophilic silica with hexamethyldisilazane (HMDS) to form bipolar silica. After coated and heated, the covalent bonds were formed between the substrate and the middle connecting layer as well as between the middle connecting layer and the bipolar top layer. The obtained superhydrophobic surface with this method possessed high static contact angle (165°) and low rolled off angle (1°). It could bear 180 abrasion cycles under 2 kPa applied pressure on 1200 CW sandpaper. The abrasion distance was 17 cm for each cycle. When the superhydrophobic surface was soaked in 3.5 wt% NaCl solution, the Cassie-baxter state could be kept for more than 20 days. The present fabrication method of durable superhydrophobic surface was simple. It required no special equipment. It provided a direction for the fabrication of promising durable superhydrophobic surfaces.

It should be noted that this approach is also applicable to coat other substrates like Cu, Al foil, fabric etc., which can be hydroxylated first by specific solutions. However, if the substrates cannot be hydroxylated such as PS film, wood etc., the mechanical interlock will play a role between the middle connecting and the substrate. Certainly the effect of mechanical interlock is weaker than the linking formed by covalent bonds. To some extent, this approach is substrate-independent. However if the substrate can be hydroxylated, the approach is ideal.
